# Retraction: Ipriflavone and Ipriflavone loaded albumin nanoparticles reverse lipopolysaccharide induced neuroinflammation in rats

**DOI:** 10.1371/journal.pone.0294102

**Published:** 2023-11-02

**Authors:** The PLOS ONE Editors

Following the publication of this article [1], concerns were raised regarding Fig 8. Specifically,

- The bands in the following lanes appear more similar than would be expected from independent results:
  ○ Fig 8A p38-MAPK: lane 2 and lane 6
  ○ Fig 8A p38-MAPK: lanes 8–9 and lanes 11–12
  ○ Fig 8A p-p38-MAPK: lane 3 and lane 9
  ○ Fig 8A β-actin: lanes 2–3 and lanes 11–12
- In all panels, when levels are adjusted to visualize the background, the signal surrounding the bands appears to be discontinuous with the rest of the panel.
- The Supporting Information files do not include the original blots for Fig 8 as per the journal’s requirements at the time of publication [2].

The corresponding author of this article stated that the original data for Fig 8 were not available for editorial review. They also stated that discontinuities in the background signal may be due to insufficient washing protocols. In the absence of the raw data required to support the published results PLOS is unable to resolve the above issues.

In light of the concerns affecting this figure and the absence of original data that question the reliability of these data, the *PLOS ONE* Editors retract the article.

NWY, DAG, and MAED did not agree with the retraction. SK, SRS, and MMES either did not respond directly or could not be reached.
